# A Culture of Consent: Legal Practitioners’ Experiences of Representing Women Who Have Been Misidentified as Predominant Aggressors on Family Violence Intervention Orders in Victoria, Australia

**DOI:** 10.1007/s10691-022-09506-5

**Published:** 2023-02-14

**Authors:** Ellen Reeves

**Affiliations:** School of Social Sciences, Monash University, 20 Chancellors Walk, Clayton, VIC 3800 Australia

**Keywords:** Australia, Court responses, Family and domestic violence, Legal practice, Law, Misidentification

## Abstract

There is currently unprecedented attention in Australia on the misidentification of women victim-survivors as family violence ‘predominant aggressors’—this focus has largely been oriented towards the role of the police. Less research has considered court responses to misidentification and specifically, the role that legal practitioners play in recognising and responding to clients who have been misidentified. This article addresses this key gap in the literature through an exploration of 18 legal practitioners’ experiences of representing misidentified clients in the civil protection order system in the Australian state of Victoria. The findings suggest that legal practitioners face a number of challenges when representing clients who have been misidentified and that the magistrates’ courts are ill-equipped to respond to misidentification. As a consequence, a culture of respondents consenting to orders that should never have been made against them is maintained. This article calls for a greater focus on the role that the courts can play in providing a ‘safety net’ for victim-survivors who have been misidentified.

## Introduction

Over the last decade in Australia, the misidentification of women victim-survivors of family violence as ‘predominant aggressors’ has received unprecedented attention (Wangmann 2009; Mansour 2014; Ulbrick and Jago 2018; Nancarrow et al. 2020; Reeves 2020, 2021). Current criminological research is particularly focused on the role of police in misidentification, notably in the civil protection order (CPO) space, where police play a pivotal role in initiating CPO applications against potential perpetrators. CPO systems have frequently been referred to as existing within a ‘quasi-criminal’ legal system, in that whilst the orders are civil, the contravention of a CPO is a criminal offence. Perhaps in no other jurisdiction are the blurred lines between civil and criminal law more apparent than in Australia’s CPO system(s). Unlike other Western jurisdictions such as the United States (US) and the United Kingdom (UK), where CPO applications are primarily sought by applicants privately, approximately 77% of CPO applications are police-initiated in Australia (see Crime Statistics Agency 2021), thus presenting a unique policy context. Further, police acting as third-party applicants in the majority of CPO matters means that they also act on behalf of affected family members (AFMs) in court, making Australia’s CPO system(s) distinct from other civil law matters. Respondents are expected to seek their own legal representation to ‘defend’ themselves against police applications, and this in many ways mirrors the adversarial criminal legal system.

A strong consideration of frontline police practice is warranted, as improving police responses in order to be more trauma-informed and context-driven is a key factor in addressing misidentification (Tolmie et al. 2018). However, despite the unique legal context outlined above, there has been minimal focus on how misidentification presents, and is responded to, in the courtroom. With legal practitioners playing a key role in supporting misidentified parties, there is a need for research that specifically considers their experiences and views on best practice in relation to seeking redress in misidentification matters.

The Australian state of Victoria presents a valuable research context to further examine how the courts are responding to misidentification in the CPO (‘family violence intervention orders’ (FVIOs) in Victoria) space. In its 2016 landmark report, the Victorian Royal Commission into Family Violence (RCFV), highlighted the issue of misidentification and made police-specific recommendations aimed at reducing the rates of its occurrence (RCFV 2016). Whilst bringing important attention to the issue and making recommendations that may support the courts in their efforts to respond to misidentification, it did not make any specific recommendations in relation to how magistrates’ courts, where FVIO applications are considered, respond to misidentification. Of importance to this article, Recommendation 77 *did* advise that the courts “develop a safe, supported negotiation process for family violence intervention orders” (RCFV 2016, 67). This recommendation saw the Centre for Innovative Justice (CIJ) undergo an investigation into the consent negotiation process, finding that the current process is unsafe and undermined by many parties’ lack of access to independent legal advice and pre-court service engagement, rendering respondents and AFMs alike often mystified by the FVIO process and the decisions made in court (Campbell et al. 2021). The report, however, focused on victim-survivors as AFMs and perpetrators as respondents. It offered little insight into how the consent negotiation process is experienced by women misidentified as predominant aggressors or the legal practitioners who represent them.

When a person has a CPO application made against them, they may consent to the order with or without formally admitting to the facts outlined in the application, or they can contest the order in further court hearings. Most orders are made by consent in Australia (ALRC & NSWLRC 2010; RCFV 2016; Campbell et al. 2021) and whilst this may be beneficial for many victim-survivors and perpetrators alike, and may reduce pressure on the court system, it raises significant concerns in regard to victim-survivors who have been misidentified and then agree to orders that should not have been made against them. Magistrates considering consent orders are to determine whether it is “in interests of justice” to make the order and if the order may pose a “risk to the safety of one of the parties or a child of the affected family member of the respondent”^1^. Despite this requirement to consider the safety implications of consent orders, the CIJ found that it is common practice for magistrates to ‘rubber-stamp’ consent order proposals, with little consideration of how consent was negotiated (Campbell et al. 2021).

This article critically examines this culture of consent from the perspective of 18 Victorian legal practitioners and offers insight into the key challenges faced by legal practitioners in representing misidentified clients and navigating a system that has not been designed to account for misidentification. The qualitative analysis of legal practitioner interviews seeks to stress the importance of the courts serving as a safety net for women who have been misidentified, especially where current policing practices are not yet to a standard where all women are adequately supported and protected through the FVIO system. The article first examines what is currently known about the misidentification of women as predominant aggressors internationally and within the Australian CPO context, including a consideration of the dearth of research on how these cases present and are responded to in the courtroom. Following this, an outline of the research methodology and limitations is offered. Interview data are then presented, with a consideration of three key themes that speak to the challenges faced by legal practitioners representing misidentified clients: the time and resource constraints on the court and its actors; the benefits and risks of misidentified clients consenting to orders; and the benefits and risks of misidentified clients contesting orders. The discussion and conclusion places emphasis on the ways in which Victoria’s FVIO system is not designed to respond to misidentification, often leaving legal practitioners and their clients in a ‘lose-lose’ situation. Greater awareness of misidentification and legal system abuse, accompanied by adequate resourcing to the court system are posited as a key solutions to the magistrates court’s current inability to act as a safety net for women victim-survivors who have been misidentified as predominant aggressors.

### The Misidentification of Women Victim-Survivors as ‘Predominant Aggressors’

Family violence is widely recognised as a significant social, political, economic and public health issue, one that effects approximately one in six women and one in sixteen men in Australia (AIHW 2019). Family violence is a gendered issue, and given this context, family violence research is largely orientated towards a dichotomised examination of experiences—that being women as victim-survivors and men as perpetrators (Larance et al. 2021). Less commonly researched are the experiences of women who are labelled as perpetrators by the legal system, as these experiences tend to be neglected in the much-needed focus on women victim-survivors and the challenges that they face in accessing safety and attaining justice. However, there is an emerging body of research that suggests that these experiences are often one and the same, in that a significant number of women who are labelled as perpetrators are the predominant victim-survivors in their relationship with their accuser or the ‘protected person’ (Miller 2005; Dichter 2013; Tolmie et al. 2018; Nancarrow et al. 2020).

Misidentification can occur in a range of legal contexts, such as criminal law (Finn and Bettis 2006; Hirschel and Deveau 2017), child protection (Reeves 2021), family law (Laing 2010; Fitch and Easteal 2017), and the CPO system (Wangmann 2009; Mansour 2014; Nancarrow et al. 2020)—the latter being the focus of the present study. Whilst women can be genuine perpetrators of family violence, and men can similarly be predominant victims, when we look at the context of women’s arrests and/or their being listed as respondents on CPOs, a complex pattern of victimisation, trauma, and gendered structural disadvantage often emerges and is closely tied to how women’s perpetration of family violence and/or use of force in self-defence or retaliation is understood—or not understood (by the legal system) (Miller 2005; Larance et al. 2021). Given these trends, misidentification has produced an important lens in how we understand and respond to women ‘perpetrators’ of family violence.

Research on misidentification is primarily focused on the role of police, and first surfaced in the 1980s and 1990s in the US, when it became evident that newly introduced pro- and mandatory arrest policies were resulting in significant unintended consequences for women victim-survivors. One such unintended consequence was that women were being arrested at alarmingly increased rates in comparison to before the policies’ introduction (Deleon-Granados et al. 2006; Dichter 2013). Pro- and mandatory arrest policies encourage or oblige police officers to make an arrest where they suspect family violence has been committed, serving to limit police discretion. Feminist researchers have argued that in limiting discretion, pro- and mandatory arrest policies adopt an incident-based approach to family violence which ultimately sees many women who use self-defence or retaliatory violence, or who do not fit the mould of a ‘true’ or ‘ideal’ victim-survivor, determined by police to be the one who should be subject to legal intervention (Goodmark 2008; Larance et al. 2019). This legacy of incident-based policing responses has continued in the US and has expanded to a number of other jurisdictions who have adopted similar policies, including the UK, Canada, New Zealand and Australia, all of which have observed trends in misidentification (see Comach et al. 2000; Hester 2012; Tolmie et al. 2018; Nancarrow et al. 2020).

### Misidentification in an Australian Context

Over the last decade, there has been an increasing focus on misidentification in Australia, particularly within the context of the CPO system (Wangmann 2009; Mansour 2014; Ulbrick and Jago 2018; Nancarrow et al. 2020; Reeves 2020, 2021). Australia differs from other western policy contexts in that the police are gatekeepers of the CPO system. Whilst applicants can seek protection directly through the courts, approximately 77% of CPO applications are police-initiated (see for example, Queensland Courts 2020; Crime Statistics Agency 2021). Whilst CPOs have been found to be an effective tool for some victim-survivors in gaining safety from a perpetrator (see Dowling et al. 2018), the police have long been criticised for failing to enforce the orders and to criminally charge perpetrators who violate the conditions of the order (Douglas 2007; ALRC and NSWLRC 2010; Goodman-Delahunty and Corbo Crehan 2016; RCFV 2016). Critiques have extended to consider the ammunition that CPOs can offer perpetrators seeking to further intimidate and control a victim-survivor as part of ‘legal systems abuse’, where perpetrators extend their abuse through the use of the law and other systems (Wangmann 2010; Douglas and Fitzgerald 2013; Douglas 2018).

Despite CPOs existing within the quasi-criminal system, whereby the respondent is criminally charged only if they breach the order, the role of police in the CPO space in Australia renders many of the issues identified in the US criminal legal system relevant in the Australian context. For instance, a key concern of US researchers is that rather than being genuinely gender-neutral, family violence policing policies may place women’s use of force within the context of men’s violence—in that police may view women’s violence the same as men’s, despite evidence that women most commonly use of force within the context of their own victimisation and/or to protect others, such as children (Miller 2005; Li et al. 2015). Alternatively, driven by incident-based responses, the police may be less concerned with the broader contexts of violence than they are with ‘who hit who’, which may place women victim-survivors who ‘fight back’ at risk of misidentification (Wangmann 2012; Nancarrow et al. 2020).

Of particular concern is the increased risk of misidentification for women from marginalised and/or disadvantaged backgrounds. First Nations women, migrant and/or refugee women, disabled women, women with mental health issues, women experiencing homelessness, and women with drug and/or alcohol dependency have all been found to be over-represented as misidentified persons (Mansour 2014; Ulbrick and Jago 2018; Nancarrow 2019; Nancarrow et al. 2020; Reeves 2020). These women may be more likely to use force against a partner, for a range of reasons including but not limited to a lack of outside support systems that may be more readily available to other women (see Douglas and Fitzgerald 2018). Additionally, discriminatory and sexist attitudes of police and other key system actors may work to frame women from these backgrounds as ‘incredible’ and untrustworthy witnesses to the violence committed against them (Ulbrick and Jago 2018; Nancarrow et al. 2020; Reeves 2020). Compounding such factors is the active role that the abuser plays in ‘framing’ the victim-survivor as the predominant aggressor, which is often achieved by manipulating police officers through tactics such as self-inflicting wounds, ensuring they are the party to call the police first and making false allegations about the victim-survivor (Nancarrow et al. 2020).

### Court Responses to Misidentification

It is well-documented that the court process for victim-survivors of gendered crime, including sexual assault and family violence, can be retraumatising and experienced as an extension of abuse (Ptacek 1999; Bell et al. 2011). Looking beyond the criminal court process, many of these issues also permeate CPO hearings—women seeking protection have reported feeling silenced by the process and having had their allegations treated with suspicion (Connelly and Cavanagh 2007; Hunter 2008).

Whilst there is an emerging body of literature on how CPOs progress through the courts generally, there is little on how key legal actors respond to instances where a person may have been incorrectly labelled as a perpetrator. Australia serves as an important focal point for such experiences, due to its heavy reliance on CPOs and the growing awareness in the judiciary of misidentification and legal systems abuse (Douglas 2018; Douglas and Chapple 2019; Nancarrow et al. 2020; Reeves 2021). Of key concern are the use of cross-applications, whereby both parties have initiated or have had initiated on their behalf by the police, CPO applications against one another. This often occurs when a woman victim-survivor has sought protection for herself, and her male abuser has then sought a retaliatory order—often with the aim of intimidating the victim-survivor to a point where she withdraws her initial application (Wangmann 2009). Indeed, Wangmann (2010) explored patterns of cross-applications in the Australian state of New South Wales, finding that there were key gender differences in men’s and women’s applications. These differences suggested that “some men’s allegations fall within a totally different category [to women’s], a category that seeks to utilize a legal mechanism as a way to challenge women’s claims for safety” (Wangmann 2010, 968), a finding which was similarly observed in US research (Durfee 2011). Douglas and Fitzgerald (2013) highlighted that where cross-applications are police-initiated, women are more likely to consent to the order than their male counterpart; however, men are more likely to have their applications dismissed or withdrawn in court, suggesting that key actors, such as magistrates, may be more likely to view men’s applications as retaliatory or as an attempt to further control the woman victim-survivor.

Magistrate responses to misidentification are inconsistent. Studies have highlighted mixed experiences of legal practitioners and victim-survivors, with some suggesting magistrates play a pivotal and supportive role in addressing misidentification, and others citing experiences with magistrates who continue to tolerate vexatious applicants and ‘nasty’ tactics used by men and the private lawyers representing them (Reeves 2020, 2021). Nancarrow et al. (2020) found that there may be a growing awareness within the judiciary of misidentification, with some magistrate participants believing that when they focus on the role of ongoing patterns of abuse, including non-physical forms of abuse, they are more likely to detect vexatious applications.

Despite evidence of improving practice, magistrates have expressed concern over the time and information limitations that they experience, which may result in parties subject to orders not fully understanding the conditions of that order, leading to contraventions (RCFV 2016). Additionally, both Nancarrow et al. (2020) and Reeves (2020) have highlighted the barriers presented by police prosecutors who may be unwilling to withdraw applications at the first mention hearing, whether they believe misidentification may have occurred or not. Police risk aversion may be viewed as a positive consequence of increased pressure on police to take all allegations of family violence seriously; however, it similarly manifests as police taking action in *all* cases to ‘cover their backs’ for fear of professional repercussions should they fail to protect someone (Meyer and Reeves 2021; Myhill and Johnson 2016). This may result in the unintended consequence of leaving victim-survivors who have been misidentified with little opportunity for redress. Duty lawyers currently operate under significant time constraints which may result in encouraging misidentified parties to consent to applications made against them (Nancarrow et al. 2020; Reeves 2020). Such a context creates an environment where legal systems abuse may flourish, with perpetrators being successful in using the CPO system as a tool of further control. For instance, when the respondent has consented to an order, the AFM is in a position where they can extend their control over the respondent through the law. They may deliberately encourage or manipulate the respondent into breaching the order and then report them to the police (Nancarrow et al. 2020; Reeves 2020, 2021).

It is pivotal that there is a continued focus on improving police responses so to minimise this risk and better protect victim-survivors; however, it is also imperative that we consider what role the court may play in providing redress in misidentification cases and operating as an effective safety net. The present study seeks to address this gap in the literature, highlighting the constrictive environment in which legal practitioners operate, whereby they are often placed in a lose-lose situation when it comes to seeking safe avenues of redress for their clients.

## Methodology

The data presented in this article was gathered over a 12-month period in 2019 and 2020 as part of a larger project on the misidentification of women as predominant aggressors in Victoria. 18 legal practitioners were recruited for interviews. All interview participants were ‘duty lawyers’, who are lawyers provided to clients by the court, and worked for legal aid or community legal centres. It is therefore important to acknowledge that the findings of this study reflect a particular kind of lawyering experience, which is notably different from that of private lawyers, who have greater resourcing capabilities. All participants were recruited and contacted through their organisation contact details, which are publicly available.

Interviews with participants were semi-structured, as this method offers many benefits to both researcher and researched, including the ability to follow-up on points raised by the participant and to examine relevant issues that had hitherto not been considered by the researcher (Skinner 2005). It also allows for greater rapport-building between the two parties, which is conducive to a more fruitful and open discussion (Kirsch 1999). Participants were asked broadly about their views on misidentification and how the system responds to the issue, in addition to their professional experiences of representing clients who they believe have been misidentified. Interviews usually ran for 30 minutes to one hour. Legal Practitioners 17 and 18 submitted written responses to the interview questions, as they were recruited at the beginning the COVID-19 pandemic, and due to the significant pressures on services at this time it was mutually decided by the researcher and the participants that this would be the most appropriate and least time-consuming way to facilitate participation in the study. The beginning of the COVID-19 pandemic coincided with the data reaching saturation point (32 stakeholders were interviewed in total), whereby no new themes were emerging in interviews and this stage of data collection was therefore concluded.

It should also be noted that 5 of the 18 participants were interviewed in focus groups, and two of these focus groups consisted of other family violence professionals, such as social workers and family violence advocates. Whilst the initial research design sought to carry out one-on-one interviews, focus groups were conducted where multiple employees of an organisation wished to participate, and a focus group was the best utilisation of staff time. The data yielded in focus groups is likely different from that which would have been procured had it been a one-on-one interview—for instance, rather than focusing on misidentification purely from a legalistic perspective, groups that involved participants with other system perspectives allowed for a more holistic picture of the issue. However, for the purpose of this study, the analysis is based only on the comments of legal practitioners.

Barriers were faced in accessing participants who specifically work for Australian First Nations specialist services, and this is a key limitation of this study. Recent research has found that First Nations women are over-represented as misidentified persons and may experience more punitive responses from the legal system when they have been labelled a perpetrator (Douglas and Fitzgerald 2018; Nancarrow 2019; Nancarrow et al. 2020). Attempts were made to recruit from these services but were unsuccessful, perhaps due to the significant underfunding of these services, which may limit their ability to engage in research projects. Another notable limitation is that this study does not consider the operation of Victorian specialist family violence courts, wherein the time and resource pressures present in court proceedings may be less pronounced (e.g., fewer matters heard in a sitting). Whilst some participants had experiences of working within specialist courts, they were not asked to differentiate these experiences from non-specialist courts.

Interviews were audio-recorded and transcribed by the researcher. Transcripts were then uploaded into qualitative data management software NVivo for coding. Transcripts were coded using thematic analysis, a flexible methodology which allows the researcher to “identify patterns within and across data in relation to participants’ lived experience, views and perspectives, and behavior and practices” (Clarke and Braun 2017, 297). All participants were guaranteed anonymity and, where quoted in this article, are assigned a numbered pseudonym (e.g., Legal Practitioner 1).

### Findings

#### Time and resource constraints of the court system

Victorian magistrates’ courts are a fast-paced environment. The RCFV (2016) found that the average number of cases on family violence lists across all Victorian magistrates’ courts is 30 per day; however, Legal Practitioner 5 stated that they have seen lists of 60–70. Family violence matters run for an average of 7.34 minutes in the courtroom (RCFV 2016, vol VII, 224) and FVIO matters are generally heard on dedicated family violence days. Involved parties are required to be at court at the beginning of the day and to wait until their case is heard. Thus, whilst they may spend the majority of the day at court, they often only spend a few minutes before a magistrate. Similarly, those who are represented by duty lawyers, rather than private lawyers, may only have a few minutes with their legal representation prior to the hearing. For some clients, this is the first time that they have met their lawyer.

Legal practitioners in this study identified the fast-paced court process as a product of under-resourcing, both for the courts and for supporting legal services. Operating within these limited parameters, legal practitioners are expected to balance safe and appropriate legal outcomes for clients, with economic and efficient ones (see Victoria Legal Aid 2020), which is increasingly challenging in cases where they believe their client has been misidentified. Time and resource strains were posited as a key barrier in adequately representing women who have been misidentified:

… so it’s often hard to get a sense of what has happened unless you’re really able to go through with the client, you know, step by step … often you’ve got really limited time to really go through what’s happened and be able to put together [a] coherent submission about why they’ve been misidentified. (Legal Practitioner 4)

As identified by a number of participants, making submissions to the court that challenge the FVIO application against their client and establishing a history of abuse perpetrated against the respondent (the client) by the AFM, is an important part of providing redress in misidentification cases. FVIO first mention hearings are largely one-sided in their operational nature and making submissions to the court may be the only opportunity to highlight the risks to the respondent. However, as emphasised by Legal Practitioner 4 above, these efforts may be undermined by the limited time that lawyers are given with a client, which creates challenges in their ability to present the ‘whole picture’ of the relationship and of the abuse. In particular, it creates challenges in capturing women’s experiences of coercive control and how these experiences often play a key role in their being determined the predominant aggressor, especially where the male perpetrator has manipulated the police and/or other system actors.

Further exacerbating the issue is a lack of access to interpreters for clients from migrant or refugee backgrounds. Migrant and refugee women face unique risks and forms of control within the context of family violence. The perpetrator may weaponise the victim-survivors’ lack of knowledge of Australian legal systems and/or their fears of having their children removed from their care or being deported if they do report family violence (Segrave 2017). Within this context, women from migrant or refugee backgrounds are at an increased risk of being misidentified. A key factor here is the victim-survivors’ fear of telling the police that they are a victim-survivor, combined with their inability to communicate this where no interpreter, or no appropriate interpreter (the police may sometimes use the perpetrator) is provided by the police—rendering these women’s experiences unheard, or heard only through a perpetrator lens (Ulbrick and Jago 2018; Nancarrow et al. 2020; Reeves 2020). Such issues appear to similarly present in the courtroom, compounding legal practitioners’ ability to represent clients in a way that accurately reflects that particular client’s experience of family violence, with a lack of time with clients exacerbating this.

Resourcing deficits were also discussed by legal practitioners in relation to magistrate practice and their ability to appropriately respond to misidentification. This is relevant, as magistrates play a significant role in “shaping the practice and culture in a court environment” (Campbell et al. 2021, 61) and whether this practice and culture will prioritise women’s safety. Legal Practitioner 1 believed that “any weaknesses that we see in the magistracy is, I think, attributed at least partly to the chronic under-funding of the magistracy system”. The effect of this, according to a number of participants, is a limited ability of magistrates to take time to recognise and respond to misidentification, instead relying primarily on the narratives presented to them in the police application, alongside the respondent’s apparent willingness to consent to the order. For example, Legal Practitioner 9 was of the view that magistrates are relatively powerless at a first mention hearing, and in regards to misidentification, they argued that “magistrates don’t have the time or space to be able to deal with these [matters]”, which may mean that they do not have the capacity to make nuanced decisions reflective of the complexities of women’s victimisation. This participant went on to suggest that it is only when a case goes to a contest hearing, of which very few do, that the magistrate can take the time to properly assess the case and provide redress when a victim-survivor has been misidentified. Whilst a first mention will not involve a testing of evidence, magistrates are required to assess to appropriateness of a consent order proposal, which the CIJ found to be occurring inconsistently (Campbell et al. 2021).

It should be noted here that whilst a number of participants questioned the power of magistrates in responding to misidentification, a similar number viewed the role of the magistrate in FVIO hearings as pivotal, describing the practice of a number of Victorian magistrates who, despite the backlog and inundated family violence lists, “can really put a stop to [misidentification]” (Legal Practitioner 12). In these instances, participants spoke about magistrates having in-depth understandings of the gendered nature of family violence and the different ways in which women victim-survivors may present. These varying views speak to the inconsistency of practice across different court locations (see also Fitzgerald et al. 2021).

The perspective of participants who believed that magistrates hold minimal power in the initial hearing is interesting when considered against the views of those who raised concerns about the ‘one-sided’ nature of an FVIO hearing. Some legal practitioners highlighted that an FVIO hearing is not designed to hear two sides of the story—it is designed to establish whether an FVIO should be made against the respondent protecting the AFM, based on evidence primarily collected by the police in relation to the respondent’s ‘offending’ behaviour. Victim-survivors, and the lawyers who represent them, are rarely given the opportunity to detail their experiences of abuse perpetrated by the AFM unless they go to a contest hearing. Legal Practitioner 13 reflected on this challenge,

“Because there’s all these really strict rules of evidence … it’s really difficult because we cannot lead evidence of family violence from the bar table, so it’s in the courtroom but we’re not the applicant.”

This raises important questions about who does hold power in these hearings if not legal practitioners or magistrates. Whilst some legal practitioners cited proactive lawyering and judicial practice, it is apparent that first mention hearings are often not an environment particularly conducive to an acknowledgement of misidentification, and more broadly, the *patterns* of power and control that characterise women’s experiences of family violence. Instead, according to participants, it may be the case that due to the huge demand on the magistrates’ courts, a demand which is not adequately met by resources, the status quo as presented by police prosecutors, is maintained. This ‘status quo’ is largely characteristic of an incident-based understanding of family violence, which takes victim-survivor and perpetrator actions out of their original context (Wangmann 2010). As argued by Legal Practitioner 5, some magistrates may “essentially just rubber-[stamp] police’s applications”. This inevitably places limitations on the ability of legal practitioners to argue a case in court and to put forth their client’s own need for protection and to challenge the validity of the FVIO application. Consequently, abusers are emboldened to commit this form of legal systems abuse.

It was also observed that police prosecutors may be becoming increasingly risk averse in the wake of the RCFV (2016), in that they are becoming less willing to withdraw applications:

It’s, you know, if the police have gotten it wrong, it’s a little bit late by the time we get to court for police to do much, unless they are willing to withdraw on the day, which as you may or may not know, with intervention order applications … it’s essentially policy that they never will. (Legal Practitioner 12)

Risk-averse policies are an inherently positive shift in the legal response to family violence, reflecting the growing awareness of its severity; however, misidentification serves as an example of where they may have harmful implications. Where police prosecutors are unwilling to seek withdrawal at a first mention, victim-survivors and the lawyers who represent them are thrust into the difficult choice between consenting to or contesting an order, which is further bolstered by the time constraints felt by the court.

#### The benefits and risks of consenting to a family violence intervention order

The above discussion highlighted the structural challenges faced by legal practitioners when representing clients who they suspect have been misidentified, challenges that are largely symptomatic of the significant time and resource limitations of the court. An additional challenge identified by legal practitioners was determining if it is in the best interests of their client to consent to an order or contest it at a later court date. As mentioned, the status quo in FVIO hearings is one of consent—when a respondent consents to an order, they are out of court relatively quickly and usually have not made an admission of fact. Additionally, consenting to an order involves a negotiation process, where the respondent may agree to the order based on lesser restrictions than originally requested in the application, thus giving them some agency in the process. Given this, it is unsurprising that approximately 70% of FVIOs in Victoria are finalised by consent^2^ (RCFV 2016, vol. VII, 42).

In addition to the benefits to the respondent (and the AFM), the culture of consent alleviates some of the pressures on the court system, with fewer respondents returning to court to dispute applications (ALRC and NSWLRC 2010; Campbell et al. 2021). The gendered operation of the law, which sees women’s allegations subject to scepticism (see Epstein and Goodman 2019), renders the court process for victim-survivors of family violence highly stressful, sometimes dangerous, and often retraumatising (Bell et al. 2011). For persons who have experienced family violence and are engaging with the court system as ‘perpetrators’, this may be increasingly so (Reeves 2021). According to legal practitioners, most clients who have been misidentified prefer to consent to the order because “they just want to get out of there and they’ve lost their faith in the system” (Legal Practitioner 13) and will therefore negotiate consent with the AFM. Continued engagement with the court process may also place victim-survivors at increased risk of abuser retaliation. Legal Practitioner 16 spoke about this specifically in relation to women from migrant or refugee backgrounds, many of whom “have of lot of fear for the law”, which may render them unwilling to engage with the court process. Additionally, whilst some respondents may not be seeking separation from the AFM, if they are, having an FVIO that limits contact between the parties may be useful, as highlighted by Legal Practitioner 8:

… you know, sometimes my advice to the client is if the practical implication of it is, that you can’t go anywhere near him or, you know, you can’t commit family violence and you don’t do those things anyway, [what] practical impact is it going to have for you moving forward? (Legal Practitioner 8)

For Legal Practitioners 8 and 9, consent was seen as the lesser of two evils in some situations, depending, of course, on the level of risk to the client.

Whilst Legal Practitioner 1 emphasised the need to take time to explain to clients the implications of consenting or contesting, thus empowering that client to make an informed decision, Legal Practitioner 16’s comments suggest that this can be challenging:

… they’ll say "“I just want it to go away”" and it’s really hard to try and explain in a short period of time… “these are the consequences, this is what you need to do, these are your responsibilities”, ask them to make a choice because, to them, they don’t want to be anywhere near a court.

When Legal Practitioner 16 refers to the “consequences” of consenting to an order, they, like a number of participants, are referring to the significant potential for the final FVIO to be used as a tool for further abuse by the perpetrator against the victim-survivor. Contravention of an FVIO is a criminal offence with a maximum penalty of two years imprisonment and/or 240 penalty units ($43,617.60)^3^ (Sentencing Advisory Council 2021). Legal practitioners spoke about male perpetrators strategically encouraging victim-survivors who have been listed as respondents to breach the FVIO against them as part of their legal systems abuse campaign, which may result in criminal charges. Legal Practitioner 1 said that “often in those circumstances the perpetrator would very cleverly...kind of lure her to breach the intervention order and she can become criminalised” and according to Legal Practitioner 9, “the danger is that perpetrators of family violence will use that tool to further manipulate, control, coerce, like they use that as a tool to further get what they want.”

These breaches might occur in a range of scenarios. For instance, as highlighted by Legal Practitioner 16, the perpetrator and the victim-survivor might reconnect and continue their relationship, but the perpetrator may later begin to re-engage in abusive behaviours towards the victim-survivor, or the two parties may have an argument, and as the order is still in place, the perpetrator may retaliate by calling the police and reporting the breach. Another scenario is where the perpetrator uses the context of children to encourage a breach. For instance, phone contact between the two parties might be allowed if they are discussing the care and welfare of the children, but a respondent might breach the FVIO conditions if they speak about anything other than the children. Legal Practitioner 16 described a scenario where the respondent and AFM were text messaging about the children but the AFM then changed the subject and began to insult and harass the respondent. The respondent replied to these messages and the AFM then reported the breach to the police. As the police are only likely to become aware of a breach if it is reported by the AFM, the AFM holds significant power over the respondent which can be seen and felt as an extension of the abuse in the relationship and is an example of legal systems abuse (Douglas 2018).

Thus, whilst consenting to an order may initially be viewed as a quick and easy way to have the matter resolved, the implications can be dire. Having a criminal record can impact on victim-survivors’ access to employment, education, support services (including housing), and it may also set a dangerous precedent in family law and/or child protection matters (Reeves 2021). Women victim-survivors are already in a place of structural disadvantage with many of these systems in terms of the gendered economic inequality that they face (Corrie 2016) and the continued punishment and pathologisation of women who raise allegations of family violence (Laing 2010). Legal Practitioner 13 also highlighted that when women victim-survivors do have these negative experiences, it may impact on their willingness to engage with the legal system for protection in the future:

And there are a number of times where I’ve had clients instruct me to agree to orders, where personally it gives me a yucky feeling in my stomach because I’ve heard from them what their situation is like and it’s just … it’s just a disappointing experience for them to feel so failed by that system and … in the future, those people who have had that experience of the system, that they, prior to that [understood the system] as being designed to protect them, is that they won’t make that report in the future.

Consenting to an order represents an understandably attractive option, and in some instances, it may indeed be an appropriate option, yet it may also be a problematic response which has the potential to result in significant harm to victim-survivors after they have been misidentified as predominant aggressors. Legal practitioners are therefore in a difficult position when supporting clients who do wish to consent to orders, as this may challenge their own views of what is best for the client as well as the ethos underpinning their legal service, which are often framed around seeking safe outcomes for clients, particularly women at risk of family violence. On the other hand, publicly funded lawyers are under economic pressure, obliged also to seek efficient, albeit appropriate, options—this pressure plays a key role in maintaining the engrained culture of consent in FVIO matters and has implications for the system’s ability to meaningfully prioritise women’s safety from family violence (Campbell et al. 2021).

#### The benefits and risks of contesting a family violence intervention order

Whilst all legal practitioners expressed hesitations about clients consenting to orders that they believed lack merit, the alternative was discussed less extensively. This may be due to the reality that few FVIO applications go to a contest hearing (RCFV 2016). As established earlier, a contest hearing may be the only time when a respondent can establish their own experiences of victimisation and challenge the perpetrator label assigned by having evidence tested in court. Additionally, if they are successful in a contest hearing, and have not had an interim order placed against them during the period between court hearings, the genuine perpetrator will not be in a position where they can use a finalised FVIO as leverage over the victim-survivor as they would if the victim-survivor had consented to the order. It also sets important precedent. The more misidentification matters resolved through contest, the greater awareness of misidentification there is in the court system, with contest being viewed as an increasingly viable and appropriate option, as was highlighted by Legal Practitioner 1. However, contesting an order presents its own sets of challenges and there is a tension between what is ‘best’ for the individual victim-survivor and what is ‘best’ for misidentification redress more broadly.

When orders are contested, the matter might be not be heard for months and/or may be repeatedly adjourned, and due to the impact of the COVID-19 pandemic, which has resulted in a significant backlog in the courts, this period has been further extended (see Campbell et al. 2021; Magistrates’ Court of Victoria 2021). Speaking of the pre-pandemic context, Legal Practitioner 8 expressed frustration at court delays, stating:

You get the contested hearing date and you’re booking, at the moment [August 2019], we’re booking things off for the 30th of January [2020], in [court]. So you’ve got an intervention order application that is meant to be about imminent risk and we’re booking it off for more than six months in advance.

As highlighted by Legal Practitioner 9, in the time between the initial hearing and the contest hearing(s), the magistrate may have placed an interim order on the respondent—meaning that the conditions that the respondent is contesting might be in place for the period of time before they can dispute the order in court. This can include an exclusion (or ‘ouster’) order, where the respondent is excluded from the family home during this period, which is likely to impact on the respondent’s access to children and may also place them in unstable or precarious living conditions (Reeves 2021)—already a significant risk for women victim-survivors of family violence (Corrie 2016). Legal Practitioner 15 emphasised the impacts of an exclusion order where the victim-survivor has mental health issues:

…people who are significantly unwell who are then kicked out of the only house they know like, yeah, we have loads of matters here at [organisation] where someone with serious mental health issues is not allowed to go back to the house, but that’s all they know. So they’re breaching it dozens of times over the next week because they don’t have anywhere else to go.

If the initial application sought was for a 12-month FVIO with these same conditions, and contesting the order means that the respondent must live under these conditions for six or more months regardless until it goes to contest, contesting the order may be viewed as an unnecessary extension of the court process. This is occurring at time when women are trying to navigate safety from an abuser, and continued engagement with the law may undermine these efforts. There are important reasons why an interim order will be imposed by a magistrate—allegations of family violence need to be taken seriously and if the police believe that the respondent poses a risk to the AFM, that AFM requires protection until the matter is resolved. Legal Practitioner 13 reflected on the logic of magistrates when making an interim order, even where they suspect that misidentification may have occurred:

…still the court may make an interim order against the respondent, just because that’s what’s before them. That’s the application before them, and they’ll think, “well, if I make an order that excludes the parties from being around each [other], then really if the respondent is saying that they need protection, then it’s going to protect them too because they’re not going to be around the affected person”. (Legal Practitioner 13)

However, such an approach still creates a window for further abuse, as, like final FVIOs, breaches of interim orders are criminally sanctionable in Victoria. Thus, when respondents do contest an order and an interim order is placed on them in the meantime, some of the same risks associated with consenting to an order are similarly relevant for contest.

Legal practitioners also expressed concern that even when their client is open to contesting an order, they cannot guarantee that it will go in their favour:

So if the client can consent to final order for six months … “well, they consent without admissions for a final order, it’s over and done within six months” or you book it off for six months for them to have their day in court with the risk of losing, you can’t advise someone that that’s a better option for them. (Legal Practitioner 9)

This is an important reflection given what is known about the gendered operation of the law, and the myriad of barriers that women face in being believed when they do raise allegations of family violence (Epstein and Goodman 2019). Whilst seeing an application through to contest might represent one possible way in which misidentification as a larger issue can be addressed, for individual clients, it may not necessarily improve their access to safety and to a just outcome, hence the dilemma faced by legal practitioners. Nevertheless, some legal practitioners, including Legal Practitioners 4 and 5, cited being successful in contest hearings where their clients were misidentified:

We’ve been fairly successful with ultimately the [FVIO] being withdrawn, usually not until sort of contest mentions stage so there’s a number of hearings and case conferences that in practice happen before police have a proper look at it and we can sort of put our arguments that it was a [misidentification] or ultimately she was acting in a self-defence sort of scenario… (Legal Practitioner 5)

Throughout interviews, a number of legal practitioners expressed the view that magistrate awareness of misidentification is increasing, further suggesting that there is a greater opportunity to see applications withdrawn or dismissed at the contest hearing—however, the retraumatising nature of the court process, particularly for women victim-survivors, means that this option is not being effectively utilised. Participants, such as Legal Practitioner 1, emphasised the importance of challenging orders where a person has been misidentified and viewed contest hearings as a key avenue that legal practitioners should be utilising in order to improve practice in this space.

## Discussion and Conclusion

Research on misidentification is primarily concerned with the role of police, shedding light on the common circumstances in which women are incorrectly labelled as perpetrators and highlighting how it can be prevented at this pivotal point of contact with the system (Ulbrick and Jago 2018). From this emerging body of research, it is concluded that policing responses need to shift away from a focus on single incidents of abuse, be better trauma- and risk-informed, and be better tailored to the experiences of disadvantaged and marginalised populations (Nancarrow et al. 2020; Reeves 2020). Such reforms will help address not only misidentification, but broader shortcomings in legal responses to family violence. However, there is a dearth of research which examines how misidentification manifests and is addressed in the courtroom. This is interesting given findings that suggest that when police make determinations as to who is the predominant aggressor, they are at times operating on the assumption that if they have made a mistake, the court will act as a safety net and provide redress (Campbell et al. 2021; Reeves 2021). Despite this assumption, there is limited research on whether the court does indeed act as a safety net for persons, particularly women, who have been misidentified.

This article has offered critical insights into how misidentification presents in the court system in Victoria, Australia. Specifically, it has examined the experiences of legal practitioners in representing women clients whom they believe have been misidentified as predominant aggressors. It was found that legal practitioners are faced with a myriad of challenges and ethical considerations borne out of working within a system that is not designed to account for misidentification and consequentially bolsters male perpetrators’ ability to use the law as a weapon. As highlighted, FVIO hearings are fast-paced and operate on the assumption of cases being relatively straightforward—the assumption being that the police will bring legitimate applications to the court and respondents will consent to the order. This is the status quo in magistrates’ courts, and it is a status quo that leaves little room for a consideration of misidentification, most notably since the respondent is rarely given an opportunity to present their own narrative and experiences of victimisation to the magistrate, unless they choose to take the matter to a contest hearing. Legal practitioners expressed a sense of powerlessness in the process, arguing that despite being in the court room, they are not the one making allegations and therefore there is little they can do in the way of defending their misidentified client. Some, however, also suggested that magistrates too are powerless at initial FVIO hearings, given the little time available to them to assess a case.

Who, then, holds the power in an FVIO hearing? Some participants highlighted that police prosecutors, who act on behalf of the AFM, often refuse to withdraw applications at first mention hearings, therefore placing misidentified persons in a position where they must consent to or contest the order. However, looking to police prosecutor practice as the sole contributor to the culture of consent fails to capture the whole-of-system resourcing issues, and how such issues are then reflected in broader practice. With awareness of misidentification growing amongst key court players (Douglas and Chapple 2019; Nancarrow et al. 2020; Reeves 2021), it may be resourcing that plays the most significant role in maintaining the culture of consent, rendering legal practitioners, magistrates and police prosecutors alike restricted in their abilities to obtain a fuller picture of the abuse and the relationship in question, instead being encouraged to focus on single incidents of abuse and to negotiate consent (Campbell et al. 2021). It should be noted, however, that there are distinct opportunities for magistrates to take greater care in ensuring that consent orders are not approved unless the magistrate is satisfied that the respondent has not been unduly pressured to consent to the order and understands the risks involved with consenting (e.g., criminal charges upon an alleged breach) as was recommended by the RCFV (2016). Given the limited time that clients have with duty lawyers, and the reality that some respondents will be unrepresented in court, the magistrate has a key role to play. That is not to say that magistrates should be undermining the decision of the respondent—misidentified victim-survivors should have the right to consent to an order if they view it in their own best interests—but instead ensuring that that respondent has adequate information to make an informed decision.

Legal practitioners are often faced with could be best described as a ‘lose-lose’ scenario when determining what is best for their client after being misidentified, both on an individual-client level, and in terms of addressing misidentification on a systematic level. They may advise their client to consent to the order, an appealing option as it means the client will be out of the courtroom relatively quickly and they will usually not have made any admission of fact in regards to the behaviours outlined in the application. Additionally, it may be that the restrictions imposed on them achieve outcomes they were already seeking—limited contact with the AFM. Yet, consenting to an order presents the very real risk that that order will be used as a tool of control and manipulation by the perpetrator, who may threaten, or actualise those threats through the reporting of contraventions—placing the victim-survivor at risk of criminalisation and further stigmatisation (Reeves 2021). Given this, a number of legal practitioners interviewed for this study felt conflicted about supporting their client in their decision to consent to the order, aware that it might result in dangerous repercussions for their women clients. Yet, also undermining these concerns is the lack of funding for legal services, whether this be Victoria Legal Aid, or the various other community and women’s legal services operating in Victoria. Funding constraints place further pressure on legal practitioners to encourage consent as not only is it the ‘easiest’ option for misidentified persons, but also the least costly and most efficient for legal organisations. Whilst those who participated in this study showed awareness of misidentification and some of the nuances associated with it, legal practitioners pressuring respondents to consent may also be reflective of poor practice and a limited understanding of family violence dynamics.

Misidentified parties may opt to contest an order at a later date in the hope that the application will be withdrawn or struck out by the magistrate. Whilst legal practitioners in this study spoke about this being a possible avenue of redress, with some being successful in having applications withdrawn at this stage in the court process, there was sense that this option is largely inaccessible and may result in further harm to the client, especially where it does not go in their favour. It is important to consider here the reasons that misidentified persons may not wish to contest an order and the ways in which this speaks to broader experiences of victim-survivors of family violence in court. A wealth of research, spanning over decades and multiple jurisdictions, has highlighted that the courtroom and the court process for women victim-survivors of family violence often serves as a source of secondary victimisation (see Meyer 2011). These experiences may be compounded for victim-survivors who have been labelled as perpetrators by the system. This nature of the court process plays a role in victim-survivors’ desire to disengage with the system at the earliest possible point. If contesting an order is to be viewed as a viable safety net for women victim-survivors who are misidentified as predominant aggressors, the system needs significant reform that signals to victim-survivors that they will be supported through this process, whether they are ultimately successful in the case or not (see Meyer and Williamson 2020). Currently, there is minimal incentive for victim-survivors to pursue this option, as was the view of legal practitioners interviewed in this study.

When women victim-survivors who are misidentified as predominant aggressors are encouraged to consent to FVIO applications made against them, a problematic status quo is maintained, and the growing issue of misidentification is unaddressed at the court level. Currently, legal practitioners are negotiating these matters within extremely limited parameters, leaving them with few appropriate options in terms of providing genuine redress that results in long-term safety outcomes, rather than ‘band-aid’ solutions, for victim-survivors who have been misidentified as predominant aggressors. There is undeniably an opportunity for magistrates to play a key role in providing redress by striking out or encouraging applications to be withdrawn before the point where legal practitioners and their clients are forced to make the difficult decision between consent and contest (Nancarrow et al. 2020); however, this opportunity is inhibited by the under-resourcing of the courts, with magistrates having to make important decisions in a limited amount of time based on a limited amount of information—thus restricted in their ability to make nuanced decisions reflective of the complexities of women’s victimisation experiences. The RCFV (2016) highlighted the need for capped family violence lists, however this research suggests that whilst this may have been achieved to some extent, the lists remain too long, with too many cases being heard a day, resulting in short hearing times that leave little room for broader contexts of family violence to be considered.

The RCFV (2016) also recommended that the courts develop safe and supported negotiation processes for FVIO matters. The CIJ has made recommendations for a triaged FVIO system and called for legal advice and pre-court service engagement to be made available to all involved parties (Campbell et al. 2021). Whilst the CIJ did not consider misidentification, it recognised that “practitioners were falling into rushed and transactional approaches which did not necessarily bring the [Family Violence Protection Act] goals of safety, risk management and accountability to the fore” (Campbell et al. 2021, 10). The authors emphasised the key role that magistrates should play in ensuring that orders are not made by consent without appropriate consideration of risk to either party—all orders should align with the principles of the Victorian legislation^4^.

The present study suggests that there are still significant issues with the FVIO process. The way that the system currently operates, conversations about, and encouragement of early withdrawal of applications is extremely difficult and has the effect of pressuring respondents to consent to final orders, some of which may have little merit. The court has the potential to be an important safety net for victim-survivors who are misidentified yet, awareness of misidentification is not enough. Key legal actors, whether that be legal practitioners, magistrates and/or police prosecutors, need to be supported by the system in their decision to challenge applications and to seek appropriate and safe outcomes for AFMs and respondents alike. This can only be consistently possible where courts are sufficiently resourced.

## Footnotes

1 Family Violence Protection Act 2008 (Vic) s78(4–5).

2 Based on hearings where the respondent was present.

3 *Family Violence Protection Act 2008* (Vic) s123(2)

4 Family Violence Protection Act 2008 (Vic)
